# Local tissue reaction and histopathological characteristics of three different bulking agents: a rabbit model

**DOI:** 10.1590/S1677-5538.IBJU.2020.0171

**Published:** 2021-02-03

**Authors:** Shabnam Sabetkish, Mohammad Javad Mohseni, Nastaran Sabetkish, Abdol-Mohammad Kajbafzadeh

**Affiliations:** 1 Tehran University of Medical Sciences Children's Hospital Medical Center Section of Tissue Engineering and Stem Cells Therapy Tehran Iran Pediatric Urology and Regenerative Medicine Research Center, Section of Tissue Engineering and Stem Cells Therapy, Children's Hospital Medical Center, Tehran University of Medical Sciences, Tehran, Iran (IRI)

**Keywords:** dextranomer [Supplementary Concept], Bulkamid [Supplementary Concept], Acellular Dermis

## Abstract

**Purpose:**

We assessed the efficacy and safety of a single injection of three bulking agents over the short- and long-term follow-ups in rabbits. Dermal and preputial matrices were compared with Deflux (DxHA) injection.

**Material and methods:**

Twenty-four rabbits were divided into three groups. Group I (n=8) underwent the injection of a lyophilized dermal matrix (LDM) beneath the seromuscular layer of the bladder wall. Rabbits in group II (n=8) were injected with lyophilized preputial matrix (LPM). Rabbits of group III (n=8) were injected with DxHA as the control group. They were followed up for 1 and 6 months after the injection. Subcutaneous injection of all bulking agents was also performed in nude mice. Biopsies were stained with LCA (leukocyte common antibody), CD68, CD31, and CD34. Scanning electron microscopy (SEM) and MTT assay were also performed.

**Results:**

Immunohistochemistry staining with CD68 and LCA revealed higher inflammation grade in LDM as compared with LPM and DxHA. Fibrosis grade was also higher in LDM both in short- and long-term follow-ups. However, no significant difference was detected in CD31 and CD34 staining between control and experimental groups. SEM analysis showed that the particle size of LPM was more similar to DxHA. MTT assay revealed that cell proliferation was similar in DxHA, LDM, and LPM. In-vivo assay in nude mice model showed more promising results in LPM as compared with LDM.

**Conclusion:**

The long-term results demonstrated that LPM was more similar to Deflux with the least local tissue reaction, inflammation, and fibrosis grade.

## INTRODUCTION

Bulking agents are widely used in the treatment of stress urinary incontinence and primary vesicoureteral reflux (VUR), as minimally invasive procedures. The management of patients with VUR has been revolutionized with the endoscopic injection of bulking agents which is a simple, less time-consuming, and high-resolution rate technique. Therefore, a variety of materials have been applied as a bulking agent since 1938, when the injection of sodium morrhuate around the urethra was first reported (1). The application of polytetrafluoroethylene was initialized in 1973 with significant improvement in stress urinary incontinence in short-terms (2). However, the injection of this material was widely abandoned because of particle migration, granuloma formation, and embolization. An ideal injectable bulking agent would have several characteristics including safety, hypoallergenic, non-immunogenic, well tolerance, biocompatibility, effectiveness, durability, and easy to administer. Additionally, minimal fibrotic in-growth and inflammatory response, acceptable wound healing, and retention of the bulking effect for prolonged periods are among other important characteristics for an applicable bulking agent (3).

Different materials including autologous fat, collagen, polytetrafluoroethylene (Teflon^®^), polydimethylsiloxane elastomer, dextranomer/hyaluronic acid copolymer (Deflux^®^), calcium hydroxylapatite, Macroplastique, polyacrylate polyalcohol copolymer (Vantris^®^), and zirconium carbon-coated beads have been long used as bulking agents for many years (4). Despite the overall high success rates reported in several studies, there are concerns about the short term follow-up of these bulking agents. Migration, reabsorption, and allergic reaction are amongst the posed problems of these early treatment options (4). A cure rate of 25-45% and an improvement rate of 25-70% were reported for long-term outcomes of most commercially available bulking agents (5, 6). An investigation for a more ideal material has been prompted because of the associated limitations of bulking agents.

The side effects of previously applied bulking agents led us to investigate the short-term effect of other tissue augmenting substances including lyophilized preputial matrix (LPM) and lyophilized dermal matrix (LDM). This study represents the first investigation to evaluate the acute and chronic inflammatory response of the bladder wall compared to the commercially available bulking agents. We hypothesized that LMP would be more similar to Deflux with the least local tissue reaction, inflammation, and fibrosis grade. To evaluate this hypothesis, this study was performed in a rabbit model.

## MATERIALS AND METHODS

### Preputial matrix preparation

The preputial tissue was obtained from 8 circumcised infants after obtaining informed consent from their parents. The work has been carried out following The Code of Ethics of the World Medical Association (Declaration of Helsinki). In addition, all procedures were performed according to the local ethical committee of Tehran University of Medical Sciences. Institutional Review Board (IRB) of Tehran University of Medical Sciences approved the study (IRB number: 27940). To remove the mucosal surface, Meezan's modified method was applied after washing the prepuce tissue with normal saline (7). The prepuce was decellularized with the previously mentioned method in our recent article (8). In brief, the prepuce was incubated in 1M NaCl at 38°C for 48 hours with its mucosal side downwards, to separate the epidermis from the dermis. Then, the epidermis was gently removed from the dermis with a forceps under the laminar hood. Hanks balanced salt solution (HBSS) was subsequently applied to the prepuce for 10 minutes at room temperature. This procedure was repeated for two times after changing the solution. Then, the prepuce was placed in 1% sodium dodecyl sulfate (SDS) in HBSS on the rotator at 60 RPM for 90 minutes at room temperature. The tissue was washed with HBSS for 20 minutes at room temperature.

Preputial tissue was placed in 1% Triton X-100 for 20 minutes and rinsed with phosphate buffer saline (PBS) for 10 minutes. The solution was then replaced with 0.05% trypsin/0.02% EDTA and incubated at 38°C for 30 minutes for removing the remaining DNA and RNA. The decellularized scaffolds were washed with PBS for 20 minutes. In the next step, the decellularized samples were freeze by storing at −80°C. We lyophilized the samples until completely dry (for about 24 hours). Then, a Wiley Mini-Mill was used to grind the dry ECM into a fine powder. We removed the collection jar with the milled ECM powder after most of the sample has come through the filter.

### Dermal matrix preparation

The skin tissue was obtained from 8 patients undergoing abdominoplasty after obtaining informed consent. The skin was decellularized with the previously mentioned method (9). In a preferred embodiment of the decellularization method, a dermis portion obtained by separation from the epidermis is immersed in a mixed solution containing 0.125wt % trypsin, 1mM EDTA, and 0.25wt % Triton X-100 and shaken at 37°C for 3 to 4 hours. This treatment removes substantially all cellular components (including cells of cutaneous appendages, vascular cells, fibroblast cells, and nervous system cells) within the dermis, and the dermis thus obtained is composed only of a dermal matrix containing collagen as the main constituent. Then, the decellularized scaffolds were washed with PBS for 20 minutes, lyophilized, and milled.

### DNA assay

Both decellularized dermal and preputial matrices were collected on dry ice and weighed. A Nanodrop 2000 (MaestroNano, USA) was applied for the measurement of DNA concentration (ng DNA/ mg tissue) after DNA extraction according to the manufacturer's protocol of the DNeasy Blood εt Tissue kit (QIAGEN, Netherlands). Since the matrix was lyophilized, the appropriate control was for lyophilized native tissues.

### Scanning electron microscopy

For evaluation of particle size in both LDM and LPM and comparison of those with Deflux (DxHA), several images with different magnifications were taken before injection. For this purpose, all bulking agents were coated with sputter gold (2nm thick approximately) by the application of a Gatan ion beam coater.

### Surgical technique

Twenty-four healthy male New Zealand rabbits weighing 2 to 2.5kg at 8-9 months of age and four nude mice were selected for surgical procedure. This study was conducted according to the local ethical committee of Tehran University of Medical Sciences. It has been carried out following the National Institutes of Health guide for the care and use of laboratory animals (NIH Publications No. 8023, revised 1978).

**Figure 1:**
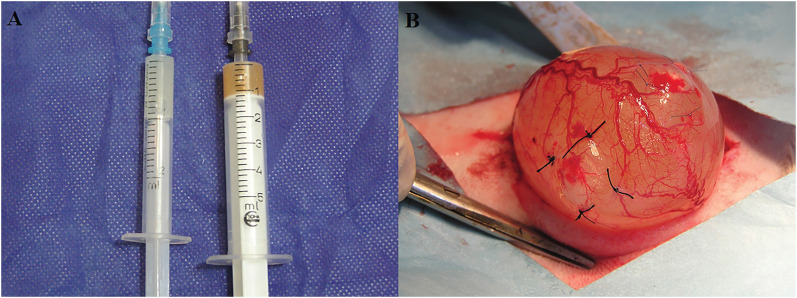
LPM and LDM supplied in the syringe at the time of injection (A) Injection method of Deflux, LPM, and LDM beneath the mucosa layer of the bladder wall (B).

Rabbit model: Intramuscular injection of Ketamine (120mg/kg) and Xylazine (15mg/kg) was applied to perform bulking agent injection under general anesthesia after suprapubic incision and exposure of the bladder. These rabbits were randomly divided into 3 groups. In group I (n=8), DxHA injection was performed beneath the seromuscular layer of the bladder as the control group. Prolene stitches were applied for marking the borders outside the bladder. The bladder was then covered with vascularized loose perivesical fat. In group II (N=8), LPM was injected with the same method. Rabbits of group III (N=8) underwent LDM injection. Injections were performed in two different sites of the bladder to be applied in different follow-ups. The mean total volume of bulking agent injected was 0.5mL per site for each animal (Figure-1). Another surgery was performed on these rabbits after 1 and 6 months for taking biopsies from the marked sections with complete bladder thickness for further histological evaluations.

Nude mice model: To evaluate the in vivo application of LDM and LPM and compare it with DxHA, intramuscular injection of Ketamine (100mg/ kg) and Xylazine (10mg/kg) was performed in four nude mice. After placing the animals in a prone position, the subcutaneous injection of each bulking agent was performed in three sites in aseptic conditions. The nude mice were kept in separate cages until 6 months after marking the injection site with non-absorbable sutures for further evaluations. Biopsies were evaluated for inflammation or any other clinical signs in the injection site.

### Histopathological evaluations

Hematoxyline-eosin (HεtE) and immuno-histochemical (IHC) staining with anti-LCA, anti-CD31, anti-CD34, and anti-CD68 antibodies were performed to evaluate the formation of fibrosis, inflammation, and angiogenesis in biopsies taken from both rabbits and nude mice. All antibodies were purchased from Dako (Trappes, France). For the fixation process, 4% paraformaldehyde was applied for 24 hours. 4μm thick slides were prepared after the blocking process. For IHC staining, the samples were immersed in Triton X-100 in 1:100 for ameliorating antibody penetration. Sections were blocked with 1% bovine serum albumin/phosphate-buffered saline (BSA/PBS) and incubated with the above-mentioned antibodies. Fibrosis and inflammation reaction was graded as follows: grade 0=no reaction, grade 1=mild reaction, grade 2=moderate reaction, and grade 3=severe reaction (10).

### In vitro evaluation

The MTT assay was performed for assessing cell metabolic activity. For MTT assay, MTT solution (3-[4.5-dimethylthiazol-2-yl]-2.5- diphenyltetrazolium bromide; thiazolyl blue) was dissolved in PBS to 5mg/mL. To filter-sterilize the MTT solution, a 0.2μM filter was applied and the solution was stored at −20°C. Subsequently, 40% (vol/vol) dimethylformamide (DMF) was prepared in 2% (vol/vol) glacial acetic acid for preparing solubilization solution. To avoid precipitation of SDS, 16% (wt/vol) SDS was dissolved in the solution and adjusted to pH=4.7. Then 10μl MTT solution per well was added to reach a final concentration of 0.45mg/mL. Afterward, it was incubated for 4 hours at 37°C and 100μl solubilization solution was added. Finally, the absorbance was recorded at 570nm.

#### Statistical Analysis

Statistical Package for Social Science software, version 15 (SPSS, Chicago, IL) was applied for statistical analysis. T-test and Bonferroni tests were applied to compare categorical and continuous variables, respectively. P <0.05 was deemed to be statistically significant.

## RESULTS

Investigation of DNA content after decellularization treatment revealed a statistically significant reduction in DNA versus the natural dermal and preputial tissues (p <0.001). Accordingly, the DNA content was significantly decreased in the decellularized dermal matrix (0.38±0.14ng DNA/mg tissue) compared to native tissue (85.76±21.12ng/mg wet dermal tissue). Similar results were obtained for decellularized and native preputial tissues (0.21±0.13ng DNA/mg tissue and 78.96±11.36ng DNA/mg tissues, respectively).

**Figure 2:**
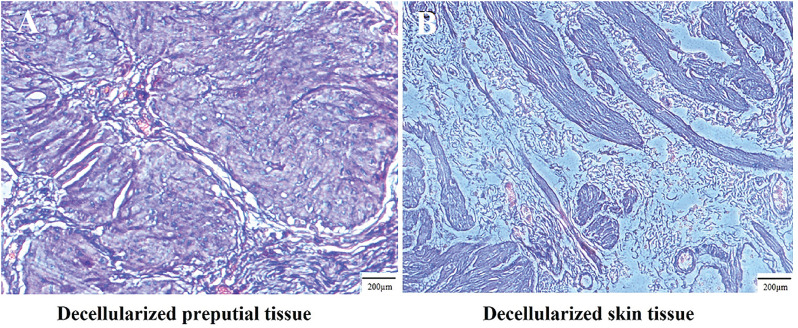
H&E staining: Decellularized preputial (A) and skin tissues (B).

**Figure 3:**
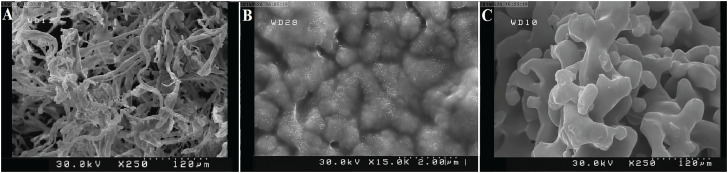
SEM analysis of LDM (A), LPM (B), and Deflux particles (C) before injection.

The results of scanning electron microscopy (SEM) revealed differences in particle size, shape, and surface characteristics in LDM, LPM, and DxHA. Larger particles with heterogeneous shapes were detected in LDM (median size of 110pm) as compared with DxHA as the gold standard bulking agent (median size of 60pm). However, the particle size of LPM (median size of 80pm) was more similar to DxHA with more homogeneity in particle shapes (Figure-2). The histological appearance of the decellularized matrices has been also depicted in Figure-3, the results of which confirmed well-preserved ECM in both matrices, suitable for achieving efficient LDM and LPM.

No intraoperative or postoperative complications such as infection, bleeding, and perforation was observed in none of the experimental models. No mortality was reported neither in rabbits nor in nude mice. No macroscopic sign of infection, necrosis, reactive changes, or allergic reaction (erythema or swelling) was noted at the time of biopsy at different time points in none of the experimental models. No distant migration was detected and all the bulking agents were placed at the same injection site in all groups.

According to microscopic evaluations in short-term follow-up, inflammatory infiltrate with lymphocyte prevalence was observed in grades 3 in LDM and reached to grade 1 in biopsies taken 6 months postoperatively. Short- term inflammation grade was 2 in LPM and reached to 0 in long-term follow-up. However, the inflammation grade was 1 in DxHA group in short-term follow-up and decreased to grade 0 in long-terms.

Regarding the fibrosis grade, histopathological evaluations revealed moderate fibrosis in LDM (grade 2) in short-terms that reached mild fibrosis after 6 months of injection. However, in LPM, grade 1 of fibrosis was changed to grade 0 of fibrosis in long-terms. According to histopathological assessments, no noticeable fibrosis was detected in DxHA group in long-and short-term follow-ups.

No significant difference was observed in the number of CD34^+^ progenitor cells in LDM and LPM in short-term follow-up as compared to DxHA group (42±0.75 and 48±0.5 vs. 52±0.25, p >0.05). Similar results were obtained for CD31^+^ microvessels (42±0. 5 and 46±0.25 vs. 50±0.75, p >0.05). The results of long-term follow-ups showed no significant difference in the above-mentioned markers either.

CD68 and LCA expressions were significantly higher in LDM as compared to DxHA in short-term follow-up (18.25±0.75 vs. 10.75±0.25, P=0.02) and (14±0.5 vs. 8.25±0.5, P=0.01). However, no significant difference was detected in LPA in comparison with DxHA in the above-mentioned IHC markers (12.5±0. 5 vs. 10.75±0.25, p >0.05) and (9±0. 75 vs. 8.25±0.5, p >0.05).

**Figure 4:**
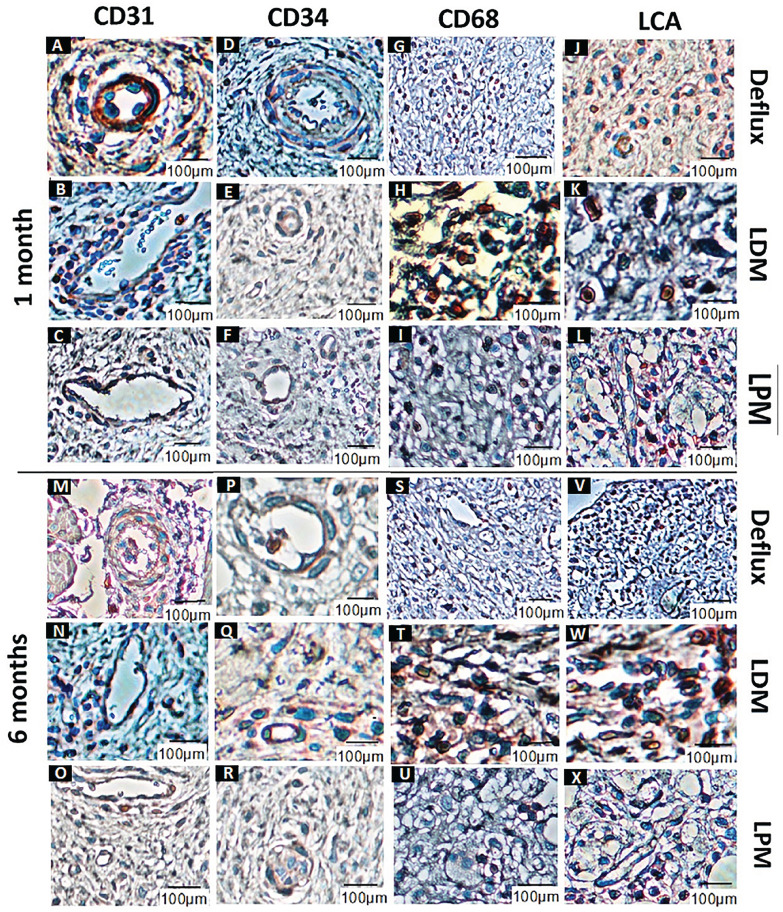
IHC staining after 1 month of follow-up: CD31 staining in Deflux (A), LDM (B), and LMP (C), CD34 staining in Deflux (D), LDM (E), and LMP (F), CD68 staining in Deflux (G), LDM (H), and LMP (I), and LCA staining in Deflux (J), LDM (K), and LMP (L). IHC staining after 6 months of follow-up: CD31 staining in Deflux (M), LDM (N), and LMP (O), CD34 staining in Deflux (P), LDM (Q), and LMP (R), CD68 staining in Deflux (S), LDM (T), and LMP (U), and LCA staining in Deflux (V), LDM (W), and LMP (X).

Even though the grade of inflammation (CD68 and LCA markers) decreased in LDM in long-term follow-up, the statistical analysis was still significantly different (10.25±0.75 vs. 5.25±0.25, P=0.02) and (7±0. 5 vs. 3.75±0. 5, P=0.01). The statistical analysis showed no difference in long-term follow-ups between LPM and DxHA (6±0.25 vs. 5.25±0.25, P=0.02) and (5.25±0.25 vs. 3.75±0. 5, P=0.01). The IHC staining for different markers is demonstrated in Table-1 and Figure-4.

**Table 1:** Immunohistochemical comparison of different bulking agents after 1 and 6 months of injection, and the results of MTT assay for in-vitro evaluation of cell viability in three different bulking agents.

	1 month				6 months				MTT assay	
	CD34 means ± SD	CD31 means ± SD	CD68 means ± SD	LCA means ± SD	CD34 means ± SD	CD31 means ± SD	CD68 means ± SD	LCA means ± SD	means ± SD of optical density (OD)	Viability (% in relation to the Deflux)
Deflux	52 ± 0.25	50 ± 0.75	10.75 ± 0.25*	8.25 ± 0.5**	85.25 ± 0.5	88 ± 0.75	5.25 ± 0.25†	3.75 ± 0.5††	0.036 ± 0.012	100
LPM	48 ± 0.5	46 ± 0.25	12.5 ± 0.5	9 ± 0.75	79± 0.5	83.25 ± 0.5	6 ± 0.25	5.25 ± 0.25	0.043 ± 0.017	99
LDM	42 ± 0.75	42 ± 0.5	18.25 ± 0.75*	14 ± 0.5**	74.75 ± 0.25	78.25 ± 0.75	10.25 ± 0.75†	7 ± 0.5††	0.054 ± 0.018	80

* P=0.02

** P=0.01

† P=0.02

†† P=0.01

Although the result of DxHA, LDM, and LPM injection revealed no significant inflammation in nude mice, moderate fibrosis was observed in the LDM group, while no sign of fibrosis was detected in DxHA and LPM after 6 months of injection.

The result of MTT showed that the means±SD of optical density (OD) in DxHA was 0.036±0.012. However, the average of OD for the LDM group was 0.054±0.018 with no significant difference as compared to DxHA. Similar results were obtained for LPM with no significant difference as compared to DxHA (0.043±0.017). The result of MTT showed that despite differences between LDM and LPM in in vivo studies, cell proliferation may be similar in the presence of LDM, LPM, and DxHA (Table-1).

## DISCUSSION

The determination of the best tissue augmenting substance is still under discussion. The outcomes of this study are remarkable in light of other published reports of synthetic bulking agents. Accordingly, both LDM and LPM had similar histopathological results as compared with Deflux as the gold standard bulking agent applied in clinical cases. However, least local tissue reaction, inflammation and fibrosis grade were obtained when LPM was applied that might be related to smaller particle size or other histological characteristics.

Deflux, as a tissue augmenting substance, has been considered as the implant of choice in the correction of VUR. The success rate after the injection of Deflux has been reported to be between 60% and 93% (11). In one study in 2012, endoscopic injection of Deflux was performed in 126 patients with VUR. Complete resolution was reported in 68% of renal refluxing units with the failure rate of 21% (12). The 6-months results of the current study also demonstrated that injection of Deflux is associated with the least local tissue reaction, inflammation and fibrosis grade.

The United States Food and Drug Administration has currently approved bovine glutaraldehyde cross-linked collagen (Contigen), autologous fat, and carbon beads/carrier gel technology (13). Numerous pilot studies have been performed in which its safety and ease of application have been underscored (14–17). Cure rates vary from 7% to 83% and if improvement rates are included in success rates, then 68% to 90% are improved or cured after collagen injection (5, 18). Even though early cure rate for a collagen injection is acceptable, the risk of allergic phenomena (4%) necessitates pre-injection collagen dermal testing which is considered as one of the limitations of its application. Minor postoperative complications have been reported and include de novo urgency that occurs in approximately 13% of patients (19), and short-lived retention in approximately 2% of patients. Although transient complete urinary retention has been reported in some studies (14, 15, 17), none of the patients encountered such a problem in the study of Corcos et al. (18). The etiology of de novo urgency is being questionable, and decreased sensation in the urinary tract may be associated with this complication (19). Occasional urinary tract infection was also reported in a previously published study (16) which can be prevented with the application of perioperative antibiotics. While few reports were published on the long-term outcome of this material, the safety and long-term outcomes (50 months) of periurethral collagen injection was reported in the study of Corcos et al. (18). However, the results of another study revealed that bovine collagen is as effective as calcium hydroxylapatite with a significant higher injection volume and repeated injections (20). One of the concerns for the application of this bulking agent is its cost-effectiveness because of the need for repeat injections. Similarly, the use of autologous fat is limited due to its re-absorption and fibrous tissue formation. According to the obtained results of a recent study (21), there is a range of complications associated with intraurethral injection of bulking agents, the most common being urinary tract infection. Therefore, in our study, we injected the related bulking agents beneath the seromuscular layer of the bladder wall with satisfactory results.

Intact human cadaveric dermal skin specimens are applied for the extraction of intact collagen fibers. After separation of the harvested dermis from the epidermis, it is mechanically powdered and disperses in solution. A buffered phosphate solution at neutral pH is used for suspension of the intact fibers. The collagen concentration ranges from 25mg/ mL (2.5%) to 100mg/mL (10%) (22). Numerous advantages are offered by this bulking agent including non-allergenicity, improved durability which is caused by natural cross-linking, and lack of tissue reaction. However, the limitation of donor supply is one of the restrictions of this material. Additionally, the injection of higher concentrations of collagen with high viscosity through smaller needles is difficult (3). Clinical studies have been conducted in ophthalmology, otorhinolaryngology, and dermatology with the application of autologous human collagen, using a similar extraction process. It has been also applied as a bulking agent for the management of VUR in animal models, the results of which revealed stability of injected volume, fibroblastic and vascular in-growth, and minimal inflammatory response (23). Our results suggested that large particles in LDM may cause more inflammation and phagocytosis after being injected, as compared with LPM. So, this aspect must be taken into consideration when manufacturing new and applicable bulking agents for clinical use.

We have previously demonstrated that preputial matrix may have potential advantages compared to other reported acellular matrix as it is easy to obtain without any need for cadaveric post mortem dissection. We also showed that the preputial matrix can be considered as a reliable source for repairing segmental urethral defects with minimal adverse reaction and excellent recapitulation of host tissue (24). Due to promising results obtained from the use of prepuce for other purposes, we tried to evaluate the characteristics of the preputial matrix as a possible material for endoscopic vesicoureteral reflux treatment.

Bioactive fibrin micro-beads embedded in cross-linked collagen has been also used as an injectable bulking agent for the regeneration of urethral sphincter muscle and treatment of urinary incontinence, with promising outcomes (25). In one study in 2017, the histopathological characteristics of polyacrylate polyalcohol copolymer (PPC) were compared with dextranomer/hyaluronic acid (Dx/HA) after injection beneath the bladder mucosa layer of rabbits. The results demonstrated severe inflammation and fibrosis in PPC due to foreign body reaction, presence of alcohol polymers, and larger particle sizes (26). Autologous chondrocytes were recently used for the endoscopic treatment of VUR in animal studies. However, the reliable application of this substance raised a serious question due to a significant high-recurrence rate after one year and the need for two different procedures under anesthesia. The patient will need to undergo the first anesthesia for harvesting the autologous cartilage cells, and the second one for injecting the endoscopic implantation (27). Therefore, the application of this substance was abandoned especially in the pediatric population.

In one study in 2019, patients underwent endoscopic correction of primary VUR by sub-ureteric injection of PPC or Dx/HA, the result of which confirmed PPC as a more effective material (28). The results of another study showed that calcium hydroxyapatite may be also applied as an effective bulking agent in the treatment of urinary incontinence in patients with bladder exstrophy-epispadias complex after injection into the bladder neck, with no significant difference in terms of bladder capacity as compared with Deflux (29).

Nude mice were applied in this study to evaluate the efficacy of using LDM and LPM in immunosuppressed patients and to compare the results with injecting Deflux as the gold standard bulking agent. Accordingly, our results revealed that the usage of LPM was more similar to the Deflux injection in terms of fibrosis formation. No signs of tumor formation were detected in the injection of LDM and LPM, confirming the usefulness of these two materials to be applied as a cost-effective substitute for Deflux. Also, nude mice were used to evaluate the cytotoxicity of different bulking agents. The long-term durability of injected bulking agents is still questionable and a subject of interest. However, the results of the current study addressed the issue of a 6-month follow-up of LPM for further clinical application. In the current study, no sign of toxicity or immunoreactivity of the biomaterials such as fat and collagen, or lack of durability of the bulking agents was observed in any of the groups. Since unforeseen long-term adverse side effects are always possible, more long-term follow-ups in bigger animals are required to investigate the efficacy of bulking agents and assess the probable migration for treatment of patients with urinary incontinence.

It should be also mentioned that this study has some limitations. The characteristic appearance of the bulking agents was not evaluated on a CT scan. Additionally, more long-term follow-ups with a larger number of animals are required to further confirm the results and evaluate the efficacy of the bulking agents for further clinical application.

## CONCLUSION

The results of the current study showed the least local tissue reaction, inflammation, and fibrosis grade by the application of LMP in rabbits. The short-term and long-term outcomes of this study are comparable to those of other established bulking agents. However, further studies are required before the widespread use of this agent.

## ABBREVIATIONS

LCA: leukocyte common antibody
VUR: Vesicoureteral reflux
LDM: Lyophilized dermal matrix
LPM: Lyophilized preputial matrix
DxHA: Dextranomer/Hyaluronic Acid
